# Antimicrobial-resistant non-typhoidal *Salmonella enterica* prevalence among poultry farms and slaughterhouses in Chitwan, Nepal

**DOI:** 10.14202/vetworld.2021.437-445

**Published:** 2021-02-20

**Authors:** Peter D. Fowler, Sumit Sharma, Dhan Kumar Pant, Subir Singh, Melinda J. Wilkins

**Affiliations:** 1 Department of Large Animal Clinical Sciences, College of Veterinary Medicine, Michigan State University, East Lansing, Michigan, 48824, USA; 2 Department of Veterinary Physiology and Biochemistry, Faculty of Animal Science, Veterinary Science and Fisheries, Agriculture and Forestry University, Rampur, Chitwan, 44200, Nepal; 3 National Zoonoses and Food Hygiene Research Centre, G.P.O. Box: 1885, Kathmandu, 44600, Nepal; 4 Department of Veterinary Medicine and Public Health, Faculty of Animal Science, Veterinary Science and Fisheries, Agriculture and Forestry University, Rampur, Chitwan, 44200, Nepal

**Keywords:** antibiotic resistance, Nepal, non-typhoidal *Salmonella*, poultry, serotype

## Abstract

**Background and Aim:**

Antibiotic-resistant infections are one of the leading threats to public health globally. Indiscriminate use of antibiotics in food animal production is an important driver of resistance, particularly among foodborne pathogens such as non-typhoidal *Salmonella enterica* (NTS). While there has been extensive research on antimicrobial-resistant (AMR) *S. enterica* in India and China, there have been few studies in countries in South Asia, including Nepal. This is particularly important with the rise of commercial poultry farming in Nepal as a means of economic development and nutritional subsistence. This descriptive study seeks to identify the prevalence and resistance patterns of NTS serotypes focusing on Chitwan, Nepal’s leading poultry producing district.

**Materials and Methods:**

A mixture of purposive and judgment sampling of 18 poultry farms and 20 slaughterhouses representing a broad geographic distribution across multiple municipalities in Chitwan was conducted in May 2019. Environmental samples taken from poultry farms included: Water, litter, feces, feed, farm swabs, and eggshell swabs. Biological samples taken from nearby slaughterhouses included: Muscle, heart, liver, skin, cecum, crop, and spleen. Samples were cultured and tested for the presence of NTS. Positive isolates were serotyped and tested for antimicrobial susceptibility to seven antibiotics known to be important to both human and animal health regionally. Farm practices were also characterized through a survey, the results of which are detailed in the accompanying paper.

**Results:**

Out of 708 samples (288 environmental and 420 biological), 103 (15%) tested positive for NTS (9% of environmental; n=26, 18% of biological; n=77). The percentage of positive environmental and biological samples varied by source. Environmental sample positive rates were water (27.5%), feces (10.6%), litter (8.6%), farm swabs (5%), feed (1.8%), and eggshells (0%). Biological sample positive rates were skin (28%), heart (23%), crop (20%), muscle (15%), liver (15%), spleen (15%), and cecum (12%). Out of 103 positive *S. enterica* isolates, 48.5% were identified as *Salmonella* Typhimurium, 35% *Salmonella* Enteritidis, 7.8% *Salmonella* Gallinarum, 4.9% *Salmonella* Virchow, and 3.9% were *Salmonella* Agona. Of the 103 positive isolates, 80 (78%) were resistant to at least one antibiotic, and 21 (20%) were multidrug-resistant (MDR).

**Conclusion:**

NTS is highly prevalent among Chitwan’s growing poultry industry with higher rates of positivity found in slaughterhouse samples compared with environmental samples from farms. In addition, a high rate of AMR (78%) was revealed, and an extremely concerning number of those were shown to be MDR (20%). This baseline data has important implications for poultry production and consumption in the region. Further research will elucidate the extent to which this contamination and drug resistance is impacting the health of the local population and help inform treatment and management strategies.

**Note:** The characterization of the poultry industry and practices that might be linked to NTS contamination in the Chitwan district are detailed in the previous paper in this series (www.veterinaryworld.org/Vol.14/February-2021/14.pdf).

## Introduction

Non-typhoidal *Salmonella enterica* (NTS) is one of the leading causes of human foodborne gastroenteritis globally, with an estimated 93.8 million cases every year resulting in an estimated 155,000 deaths [1]. This threat is compounded by the rise of antimicrobial resistance (AMR), particularly among poultry farms where inappropriate use of antibiotics as prophylaxis and for growth promotion has become widespread [2-4]. Many of these NTS serovars are frequently found to be multidrug-resistant (MDR), meaning they are resistant to three or more antibiotics which each have a unique mechanism of action, resulting in fewer treatment options, higher hospitalization rates, as well as longer recovery times [5]. Every year antibiotic-resistant infections are responsible for an estimated 700,000 deaths globally [6,7].

Nepal’s poultry industry has been rapidly expanding in the last decade, with an annual growth rate between 17% and 18% and a 261% increase in meat production from 2008 to 2018 [8]. Increased production has been met with increased consumption of poultry, rising from 10.2 kg per capita in 2002 to 12.2 kg per capita in 2011 [9]. In Nepal, poultry farming is important for both economic growth and food security. This growth has been met with many challenges such as bacterial, viral, protozoal diseases, and a reduction in effectiveness of medications for the treatment of these diseases [2].

The primary reservoir for *S. enterica* is the gastrointestinal tract of humans and animals, particularly poultry, and swine. Contaminated meats, mainly from avian and livestock origin, are the primary sources of human salmonellosis [10]. The few studies that have been conducted to date have shown a high rate of NTS contamination among Nepal’s poultry farms and slaughterhouses [11-14]. The full extent to which this impacts public health is difficult to assess due to under-reporting of clinical cases in humans and a lack of adequate surveillance data. Although there is extensive data globally on Salmonellosis and AMR, actionable data are lacking in many countries in South Asia [1]. To date, there have been very few studies on the prevalence of AMR in NTS isolates collected from poultry in Nepal.

This study aims to provide baseline data about the prevalence of NTS and characterize the AMR patterns and serotypes of the NTS isolates collected from poultry farms and slaughterhouses in the Chitwan district of Nepal. This data can immediately help guide policies to improve antibiotic stewardship among farmers in the district. In addition, this data will lay the groundwork necessary to assess resistance patterns of AMR-NTS in poultry, the serotypes present and the potential impact on human health in the region. Serotype information will help identify strains of *S. enterica* present among poultry farms and slaughterhouses that may be linked to human pathology.

## Materials and Methods

### Ethical approval

All locations were recorded at the village level to preserve anonymity of the participating facility. Sample collection did not involve handling or sampling live animals. This protocol underwent human subject review at Michigan State University and was determined not to be research involving human subjects; thus, approval from an institutional review board was not required.

### Study area and period

**Figure-1:**
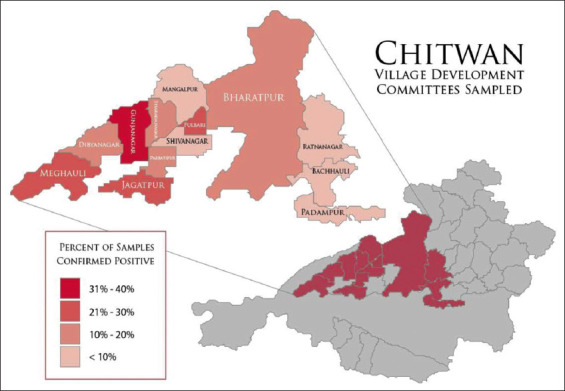
Village Development Committees sampled showing percentage of confirmed positive samples. Geographic borders are based on pre-2017 GIS data for Village Development Committees. In 2017, Village Development Committees were dissolved and replaced with Rural Municipalities, merging many areas. GIS data retrieved on 10/02/2020 from https://data.humdata.org/dataset/administrative-bounadries-of-nepal.

This descriptive cross-sectional study was conducted in the Chitwan district, which is in the southwestern part of the Narayani Zone, Central Development Region of Nepal. Villages within Chitwan were reclassified in 2017 and many borders were changed as former Village Development Committees (VDC) were changed to Gaunpalika (rural municipalities). For spatial analysis, only GIS data from former VDC was available, and locations of each sample site were recorded at this level (Figure-1). All samples were collected during May 2019.

### Data collection and sampling

Eighteen poultry farms and 20 slaughterhouses were selected across Chitwan, Nepal’s leading poultry producing district. After consulting with regional experts about the composition of the poultry industry, a combination of purposive and judgment sampling was used for the selection of sites to ensure adequate representation and geographic distribution. Farm and slaughterhouse locations were recorded by the VDC borders (pre-2017) in which the samples were taken to preserve the anonymity of the participants but still record geographic distribution of isolates among the area sampled. This included sampling farms of different sizes and types, and slaughterhouses in proximity to those farms that are representative of regional distribution of poultry and related products. Farms were selected to represent varying farm sizes: Eight large (>1000 birds), six medium (501-1000 birds), and four small (200-500 birds) farms. Among these, there were ten broiler farms and eight layer farms at various stages of production with variable types of housing. Some farms had three-sided closed concrete walls with little ventilation, and some were openly ventilated on all sides. Most housing material was similar, with a concrete floor and 61 cm (24 inch) base wall of concrete that was continued as chain link fencing to the roof. Roof material was mostly corrugated steel, often covered with thatch. Samples were taken from a variety of different sources in contact with the poultry, with larger numbers of samples taken at larger facilities to ensure adequate representation of the area. An excess of 25 g was collected for each sample type except surface swabs. For swab samples, a sterile cotton swab was dampened in 25 g of sterile distilled water and rolled across surfaces in contact with poultry such as fencing, walls, and other surfaces within the poultry farm. The area swabbed was approximately 10 cm^2^ per swab for environmental samples and three different eggs for egg samples. After swabbing, the end of the swab was broken off into the sterile water container and the container was sealed. A survey was administered to farmers to establish risk factors for NTS contamination based on risk factors identified by similar studies [15,16]. The breakdown of the survey results, sample types, contamination rates, and risk factors can be found in the accompanying paper in this series.

Twenty slaughterhouses were selected from 13 VDCs in proximity to the sampled farms. Three samples of each tissue type (crop, heart, cecum, liver, muscle, skin, and spleen) were aseptically collected from each slaughterhouse for a total of 420 samples. For muscle, liver, and skin, an excess of 25 g was collected. Crop, heart, cecum, and spleen weight were typically between 9 and 15 g, depending on the chicken. Collected samples were placed in separate sterile containers and placed in a cooler with ice for transportation to the laboratory at Forestry and Agriculture University. Slaughterhouses selected for sampling varied slightly in size, but were similar in structure, composition, and clientele, serving as a local source of poultry meat for village residents. A total of 708 (420 slaughterhouse and 288 environmental) samples were collected for testing.

### Testing: Isolation and identification of NTS

All samples were pre-enriched in 9:1 buffered peptone water for 18-24 h, before transport to the National Zoonoses and Food Hygiene Research Center (NZFHRC) laboratory in Kathmandu for further analysis. All samples were subjected to selective culture and biochemical testing for the presence of *S. enterica*. Pre-enriched samples were inoculated in HiMedia^®^ (HiMedia Laboratories, India), Rappaport-Vassiliadis (RV) broth at 41^o^C for 24 h and HiMedia^®^ (HiMedia Laboratories) *Salmonella Shigella* (SS) agar was used for selective culture. Suspected colonies were sub-cultured and isolated colonies were cultured on nutrient agar slants for further identification and biochemical characterization. Biochemical identification was done using Gram’s stain and oxidase test; all isolates showing Gram’s stain positive and/or oxidase-positive were discarded. Then, other isolates were biochemically tested using Indole, Methyl red, Voges–Proskauer, Citrate utilization, Triple sugar iron (TSI), and urease tests as per the protocol described byEwing [17]. The colonies showing Salmonella specific IMViC pattern (− + − +) were further inoculated on TSI slants, and colonies that produced alkaline slant (pink) and acidic butt (yellow) with or without H2S production (blackening) were tested for urea hydrolysis on urea agar slants. All urease negative isolates were considered as biochemically confirmed *S. enterica* spp. isolates. All media and biochemical testing reagents were manufactured by HiMedia^®^ (HiMedia Laboratories).

### Antimicrobial susceptibility testing and serotyping

Antimicrobial susceptibility testing was performed on all NTS positive cultures using Kirby–Bauer disk diffusion method outlined by the Clinical and Laboratory Standards Institute [18]. Antibiotics were selected to represent a broad range of classes which are commonly used in the region in both human and animal medicine. Antibiotics tested include: Ampicillin (AMP), gentamicin, chloramphenicol (C), ceftazidime (CAZ), cefotaxime (CTX), ciprofloxacin (CIP), nalidixic acid (NA), colistin (CL), doxycycline (DO), and enrofloxacin (EX). Serotyping of biochemically identified *S. enterica* isolates was performed according to Kauffman-White Le scheme, based on O surface antigen using group specific antisera A-G+Vi and H antisera (SII-Diagnostic, Denmark).

### Statistical analysis

Microsoft Excel 2016 ^®^ was used for data entry and management and IBM SPSS v25 was used for data analysis. The map of the sample area was created with ArcGIS v10.3.1 using administrative borders from Humanitarian Data Exchange v.1.43.5 (available at https://data.humdata.org/dataset/administrative-bounadries-of-nepal) and edited with Adobe Illustrator v16.

## Results

### Positivity by VDC

Combined results from both farm and slaughterhouses showed varying rates of positivity among the 13 VDCs. Gunjanagar had the highest percentage of samples positive for NTS at 34%, followed by Fulbari and Jagatpur which had 26% of the samples testing positive. The percentage positive for the other VDCs sampled is as follows; Meghauli: 21%, Didyanagar: 18%, Bharatpur: 14%, Parbatipur: 13%, Sharadanagar: 10%, Bachhauli: 7%, Mangalpur: 6%, Ratnanagar: 3%, and Padampur and Shivinagar with no positives (Figure-1).

### Farm samples

Of 288 environmental samples from 18 poultry farms, 26 (9%) were positive for NTS. This included 25% of all water, 11% of all feces, 9% of all soil/bedding, and 5% of all farm swabs. All egg swabs were negative for NTS. Focusing on AMR, 19 (73%) of samples positive for NTS were resistant to at least one antibiotic. Of the resistant samples, 6 (32%) were MDR. Despite the small sample size, water sampling revealed the highest rates of NTS positivity, AMR and MDR, making on-farm water sources a focal point for future studies (Table-1).

**Table-1:** Farm environmental NTS sample positivity and resistance results.

Sample type	Total number collected	No. (%) Positive of total samples	No. (%) AMR positive from total positive samples	No. (%) MDR positive from total AMR samples
Egg Swab	28	0 (0)	-	-
Farm Swab	40	2 (5)	1 (50)	0 (0)
Feces	66	7 (11)	5 (71)	1 (20)
Feed	56	1 (2)	1 (100)	0 (0)
Soil/Bedding	58	5 (9)	4 (80)	0 (0)
Water	40	11 (25)	8 (73)	5 (63)
Total	288	26 (9)	19 (73)	6 (32)

### Slaughterhouse samples

Of the 420 biological samples from 20 slaughterhouses, 77 (18%) were positive for NTS: Including 17 (28%) skin, 14 (23%) heart, 12 (20%) crop, 9 (15%) muscle, 9 (15%) liver, 9 (15%) spleen, and 7 (12%) cecum samples. Focusing on AMR, 61 (80%) of the samples positive for NTS were resistant to at least one antibiotic. Of the resistant samples, 15 (25%) were MDR. The source of AMR and MDR samples were relatively evenly distributed from all biological sample sources (Table-2).

**Table-2:** Slaughterhouse sample NTS positivity and resistance results.

Sample type	Total number collected	No. (%) Pos. from total number of samples	No. (%) AMR positive from NTS positive samples	No. (%) MDR positive from AMR positive samples
Cecum	60	7 (12)	5 (71)	2 (40)
Crop	60	12 (20)	8 (67)	4 (50)
Heart	60	14 (23)	10 (71)	1 (10)
Liver	60	9 (15)	7 (88)	3 (43)
Muscle	60	9 (15)	6 (67)	1 (17)
Skin	60	17 (28)	16 (94)	3 (19)
Spleen	60	9 (15)	9 (100)	1 (14)
Total	420	77 (18)	61 (80)	15 (25)

### Antibiogram of positive NTS isolates

**Figure-2:**
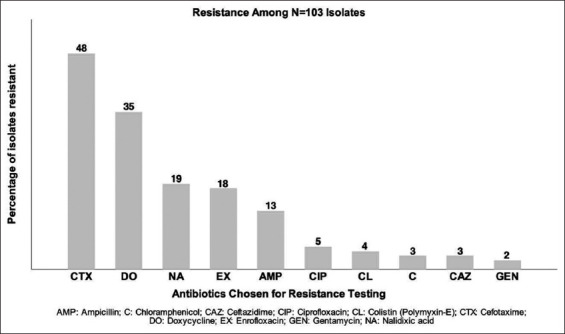
Number of 103 positive non-typhoidal *Salmonella* isolates resistant to selected antibiotics.

Of the 103 positive NTS isolates, 77 (78%) were resistant to at least one antibiotic tested and 23 (22%) were fully susceptible. Isolates showed the most frequent resistance to CTX, with 48 (47%) of the 103 positive isolates showing resistance, followed by DO: 35 (34%), NA: 19 (19%), EX: 18 (18%), AMP: 13 (13%), CIP: 5 (5%), CL: 4 (4%), CAZ: 3 (3%), C: 3 (3%), and gentamycin: 2 (2%) (Figure-2).

**Figure-3:**
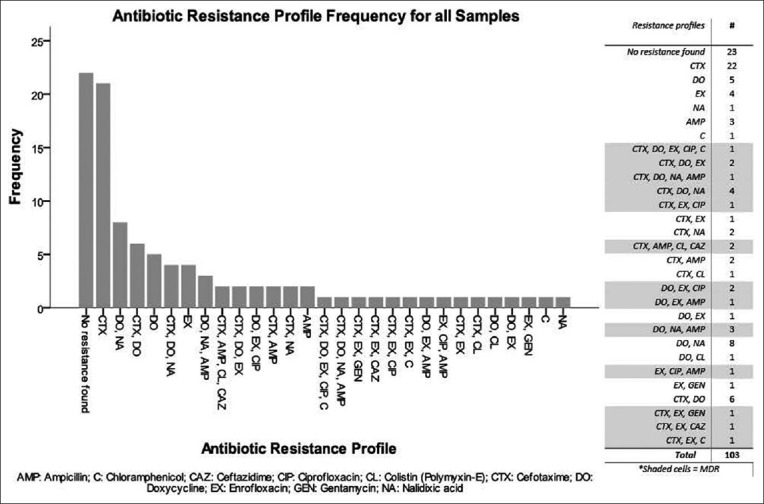
Frequency of each individual antibiotic resistance pattern observed among 103 total isolates.

The most common antibiotic resistance pattern among MDR isolates was CTX, DO and NA with four isolates showing this resistance pattern. A full list of patterns and frequencies is shown in Figure-3.

### Serotyping results

The 103 NTS isolates comprised five different *S. enterica* serotypes. *S*. Typhimurium accounted 50 (49%) of the isolates, followed by *S*. Enteritidis: 36 (35%), *S*. Gallinarum: 8 (8%), *S*. Virchow: 5 (5%), and *S*. Agona: 4 (4%). Regional distribution of serotypes is shown in Table-3. The VDC of Gunjanagar had both the highest number of positive samples as shown in Figure-1 as well as the largest variety of serotypes comprising at least one of each of the five identified, while this region makes up only 11.3% of the total samples collected.

**Table-3:** Total number of NTS by serotype and village development committee*.

NTS Serovar	Bharatpur	Gunjanagar	Fulbari	Meghauli	Jagatpur	Mangalpur	Dibyanagar	Sharadanagar	Bachhauli	Parbatipur	Ratnanagar	Total #	AMR No. (%)	MDR No. (%)
Typhimurium	12	10	4	3	6	3	4	4	2	1	1	50	41 (82)	11 (22)
Enteritidis	4	14	6	4	2	2	1	1	2	0	0	36	24 (80)	4 (13)
Gallinarum	2	1	1	0	1	3	0	0	0	0	0	8	7 (88)	3 (38)
Virchow	0	1	0	2	0	0	2	0	0	0	0	5	4 (80)	2 (40)
Agona	1	1	0	2	0	0	0	0	0	0	0	4	4 (100)	1 (25)
Total	19	27	11	11	9	8	7	5	4	1	1	103	80 (78)	21 (20)

* VDC Padampur and Shivinagar had no positive samples and are not included above.

## Discussion

Antibiotic-resistant pathogens are a major public health concern in Nepal. Within Nepal, gastrointestinal pathogens are the third leading cause of mortality for children under 5 years of age [4]. The use of antibiotics against severe cases of gastroenteritis can be lifesaving, but as antibiotic resistance increases among these pathogens, treatment options become scarce and expensive. Understanding the sources of pathogens as well as their antibiotic resistance patterns is important for mitigating transmission and updating treatment protocols. Understanding the source of exposure to pathogens (especially resistant pathogens) provides some insight into antibiotic use and misuse in the region.

This cross-sectional descriptive study found AMR NTS to be prevalent among the poultry farms and slaughterhouses of Nepal’s leading poultry producing region Chitwan. The percentage of NTS positive samples was 9% for farm samples suggesting that poultry producers in the region are making reasonable efforts to control NTS on their farms when compared with the previous studies. A 2005 study from the proceedings of the national poultry expo reported isolating *S. enterica* from 59% of environmental samples consisting of litter, food, and water [19]. Improvements in farm biosecurity protocols or vaccination practices may account for the lower rate of positivity in our study as positivity rates varied drastically between farms and VDCs with some farms in the current study having 30% of the samples positive for NTS. Another factor that may explain the discrepancy is the time of sampling. The window during which the current study was conducted was during the dry period when many facilities were at reduced production due to heat. Although *S. enterica* is known to survive for extended periods in the environment, viability and spread of the bacteria are higher in environments of high moisture, which would have coincided with the monsoon season [20] and this may have reduced viability for environmental *S. enterica* survival. There were variations in poultry housing construction materials, and housing conditions which are discussed at length in the accompanying paper. Although it was observed that houses with less ventilation had higher rates of contamination, there was no statistically significant difference, likely due to the small sample size of 18 farms.

Positivity rate for NTS of slaughterhouse samples was twice as high (18%) as environmental samples which is consistent with previous studies in Nepal. Poultry samples from retail meat shops in Chitwan revealed a prevalence rate of 46.2% among poultry samples [12]. Bhandari *et al*. [12] had a much smaller sample size and area of study which included only 26 samples from 17 slaughterhouses spanning 4 VDCs in Chitwan compared to this study which sampled from 13 different VDCs . Slaughterhouses within the current study varied drastically in the number of samples positive at any given slaughterhouse, with between zero and 48% of samples positive for NTS. In addition, the sample type is not described by Bhandari *et al*. [12], and within our sample types, the percentage of NTS positives varies from 12% for cecum and spleen to 28% for skin. A more recent study from 2017 by Shrestha *et a*l. [13] showed samples taken from slaughterhouses to have an NTS prevalence of 26.2%. The Shrestha *et al*. [13] study only used muscle and skin for sampling and shows a much closer percentage to the results of the current study which shows 28% of skin samples positive for NTS. Shrestha *et al*. [13] also had a relatively small sample size with 38 samples and an undescribed geographic distribution of slaughterhouses.

It is interesting to note the sample types that tested positive. Within the environmental samples, water represented the highest number of positive samples, and within the slaughterhouse samples, skin had the highest number of positives. One possible reason could be contamination of the water source. This may also account for the high rates of contamination of skin in the slaughterhouse samples when compared with cecum, where contamination would be expected if the chicken itself was the source. The practice of slaughter onsite before sale involves boiling the chicken before placing the chicken in the plucker and rinsing the feathers through the tumbler with water. A previous study in Chitwan [21] showed high rates of coliform contamination (68.2%), especially in water from hand pumps. Vishnu *et al*. [21], however, did not specifically look for NTS. A study in 2007 found NTS to be prevalent in potable water supply in Kathmandu with the dominant serotype being *S*. Typhimurium of which 25% were shown to be resistant to ceftriaxone, another third-generation cephalosporin [22]. An alternative explanation could be that a small number of shedding birds converging at the slaughterhouses could result in contamination of the environment and consequently other birds at slaughter. At the farm level, the insufficient cleaning of water feeders may provide a more suitable habitat for NTS growth and spread during the dry season when samples were collected. Further study of water source as a possible route of contamination is warranted.

The current study shows a far higher rate of contamination than a similar study focusing on commercial layer farms in India. In 2015, Saravanan *et al*. [23] found 1.73% of 1215 biological and environmental samples positive for *S. enterica*. Among the biological samples, liver showed the highest rate of *S. enterica* contamination at 4.8%. The positivity rate in our study was higher for both environmental samples from farms and for biologic samples, although our biologic samples were collected from slaughterhouses and not on-farm as in the Saravanan *et al* [23] study so a direct comparison cannot be made.

Regionally, the number of positives was highest in the VDC of Gunjanagar with 34% of the samples from both slaughterhouses and farms testing positive. In addition, this region showed very high rates of AMR with 85% of the isolates found to be AMR, but only 15% found to be MDR. Gunjanagar also represented the broadest number of serotypes as shown in Table-3, likely due to the higher number of positive isolates. The 80 samples taken from Gunjanagar represent 11.3% of the total samples taken, compared to Mangalpur (19.2%) and Bharatpur (18.9%). Mangalpur showed to have the lowest rate of NTS positive samples with only 6% of the 136 samples testing positive. However, among these positive isolates, 88% were AMR and 50% were MDR and comprised three different serotypes, including Typhimurium, Enteritidis, and Gallinarum. While differing terrain, population density and physical size makes direct comparisons between VDCs difficult, this may provide some insight into the presence and geographic distribution of differing pathogens. In addition, the increased amount of MDR found within some VDCs may suggest differing levels of antibiotic stewardship among poultry rearing facilities.

Among the five serotypes isolated, Typhimurium (49%) and Enteritidis (36%) were the dominant serotypes and both are of concern to public health. The percentage of Enteritidis is comparable to a recent study of raw meat samples in Pokhara Nepal, which isolated *S*. Enteritidis in 25% of poultry samples. This study found that the dominant species was *Salmonella* Typhi, the causative agent of typhoid fever [14]. The number of isolates in the Laxman Bahadur *et al*. [14] study was low (eight isolates) when compared to the current study (103 isolates). In the current study, *S*. Gallinarum was found at a much lower rate than Typhimurium and Enteritidis, representing only 8% of the isolates. However, the high rate of AMR (88%) for Gallinarum is of concern to poultry health in the region as this pathogen is the cause of Fowl Typhoid, a poultry disease with a high mortality rate (up to 100%) in susceptible flocks [24].

Antibiotic use within Nepal is largely unregulated and use within animal medicine does not require veterinary oversight or consultation. In addition, there are too few veterinarians in Nepal to serve the growing agriculture industry. Many poultry farms rely on para-vets for advice on medication. While para-vets have medical training, their training is less extensive than veterinary education in Nepal and may not emphasize the importance of antibiotic stewardship. As the poultry industry grows, so does the use of antibiotics. Between 2004 and 2005 veterinary drug use increased by 35% according to a study by Pharmaceutical Horizon of Nepal. Between 2001 and 2002 an estimated 9403 kg of antibiotics were consumed within the Nepali veterinary industry. Tetracyclines accounted for a vast majority of these antibiotics [4]. Guidelines have been put forward by the Ministry of Livestock Development attempting to limit the use of antibiotics in growth promotion within the food animal industry in Nepal in 2014. However, the lack of regulatory oversight of the sale and distribution of antibiotics makes enforcement of this policy difficult [4].

The antibiotic resistance patterns revealed in the current study show that resistance to cephalosporins is more prevalent than resistance to tetracyclines. Of particular concern is the number of isolates that were resistant to CTX, a third-generation cephalosporin of importance to human health in the area [25]. While DO is used extensively in Nepal’s poultry industry for both growth promotion and treatment of bacterial diseases, third-generation cephalosporins such as CTX are cost-prohibitive and more commonly used in human medicine. The drivers for the high levels of resistance to CTX observed on both poultry farms and slaughterhouses are unclear. While a lack of antibiotic stewardship may play a part there may be other factors. CTX-resistant bacteria have been found in other studies where no CTX was in use on the farm and a natural colonization process is suspected [26,27]. Further research may help elucidate the genetic factors leading to this resistance and is warranted as extended-spectrum beta-lactamase prevalence has been increasing among AMR and MRD pathogens in the region [13].

Nepal does have an AMR surveillance system for human illnesses established by the Ministry of Health in 1998, and now run by the Epidemiology and Disease Control Division within the Ministry of Health. However, this network does not extend to veterinary medicine, which saw a 50% increase in antibiotic sales between 2008 and 2012 [25]. Through the lens of a One Health approach, which seeks to unite human medicine with animal medicine and environmental health, the regulation of antibiotic use in animal medicine has direct implications for human health through the increased resistance of foodborne pathogens to vital antibiotic therapy. Extending this network to initiate routine surveillance for AMR pathogens from livestock and implementing regulatory oversight of antibiotic sales and use within the agriculture industry will protect human health, protect animal production and health, and prevent AMR reservoirs from developing in the environment.

## Conclusion

Nepal’s poultry industry is evolving from individual back yard flocks toward a commercial model to serve its rapidly growing urban populations. The higher rates of NTS positivity and resistance found in slaughterhouse samples versus on-farm samples is concerning, as people will increasingly be exposed to contaminated poultry from slaughterhouses as they purchase rather than raise their own poultry. Poultry represents an important source of both food security and economic security for a growing number of Nepalese. To ensure the success of this industry and the continued health of the population, it is important to understand the risks posed by foodborne pathogens such as NTS. It is also important to understand and regulate the use of antibiotics within this industry. Establishing good antibiotic stewardship, through collaborative education efforts among veterinarians, para-vets, and farmers could help mitigate the resistance of pathogens to antibiotics and improve the ­efficacy of treatment. Regulatory oversight of water quality, antibiotic sales and use, animal slaughter practices and facility hygiene, along with consumer education about food safety are critical components to control the spillover of drug-resistant strains of NTS from poultry to humans.

## Authors’ Contributions

PDF and SS1 actively worked on the sample collection, questionnaire design and initial sample processing, and draft writing. DKP supervised sample handling and completed the process of identification and isolation of *Salmonella* strains. PDF and MJW participated actively in study design and data analysis, while SS2 contributed in part to the study design, draft writing, data interpretation, and revising along with SS1, PDF, and MJW. SS2 and MJW carefully monitored each level of research. All authors read and approved the final manuscript.
